# Enzymatic Degradation of *p*-Nitrophenyl Esters, Polyethylene Terephthalate, Cutin, and Suberin by Sub1, a Suberinase Encoded by the Plant Pathogen *Streptomyces scabies*

**DOI:** 10.1264/jsme2.ME19086

**Published:** 2020-02-27

**Authors:** Raoudha Jabloune, Mario Khalil, Issam E. Ben Moussa, Anne-Marie Simao-Beaunoir, Sylvain Lerat, Ryszard Brzezinski, Carole Beaulieu

**Affiliations:** 1 Département de Biologie, Université de Sherbrooke, Sherbrooke (QC), J1K 2R1, Canada

**Keywords:** actinobacteria, common scab, cutinase, esterase, potato

## Abstract

The genome of *Streptomyces scabies*, the predominant causal agent of potato common scab, encodes a potential cutinase, the protein Sub1, which was previously shown to be specifically induced in the presence of suberin. The *sub1* gene was expressed in *Escherichia coli* and the recombinant protein Sub1 was purified and characterized. The enzyme was shown to be versatile because it hydrolyzes a number of natural and synthetic substrates. Sub1 hydrolyzed *p*-nitrophenyl esters, with the hydrolysis of those harboring short carbon chains being the most effective. The *V*_max_ and *K*_m_ values of Sub1 for *p*-nitrophenyl butyrate were 2.36 mol g^–1^ min^–1^ and 5.7 10^–4^ M, respectively. Sub1 hydrolyzed the recalcitrant polymers cutin and suberin because the release of fatty acids from these substrates was observed following the incubation of the enzyme with these polymers. Furthermore, the hydrolyzing activity of the esterase Sub1 on the synthetic polymer polyethylene terephthalate (PET) was demonstrated by the release of terephthalic acid (TA). Sub1 activity on PET was markedly enhanced by the addition of Triton and was shown to be stable at 37°C for at least 20 d.

Streptomycetes are Gram-positive bacteria that are known for their ability to produce a wide range of secondary metabolites and for the complexity of their morphological development. Although most streptomycetes species are saprophytic soil inhabitants, some are plant pathogens. Among them, *Streptomyces scabies* is the predominant causal agent of potato common scab and causes important economic losses in Canada (Hill and Lazarovits, 2005), as well as in most potato growing areas. Common scab is characterized by corky lesions on the surface of potato tubers. Similar to other soil-inhabiting streptomycetes, *S. scabies* produces a large variety of extracellular enzymes, including various glycosyl hydrolases and esterases (Komeil *et al.*, 2013; Beaulieu *et al.*, 2016). These enzymes may participate in pathogenesis because the penetration of *S. scabies* into host plants is considered to be facilitated by the secretion of extracellular cell wall-degrading enzymes (Beauséjour *et al.*, 1999).

The potato tuber is covered by a periderm that is composed of three types of tissues: phellem, phellogen, and phelloderm (Tyner *et al.*, 1997). The wall of phellem cells impregnated with suberin, a plant polymer recalcitrant to bio-degradation, is composed of a polyaromatic domain covalently linked to a polyaliphatic moiety (Bernards, 2002). The polyaromatic domain, a lignin-like structure, consists of a hydroxycinnamic acid-derived polymeric matrix (Bernards and Lewis, 1998). The polyaliphatic domain shares structural and chemical similarities with cutin, another polyester component of plant cuticles. Cutin and suberin both act as physical barriers against plant pathogens (Khatri *et al.*, 2011). Cutin and suberin are polymers of fatty acid derivatives linked by ester bonds. Cutin is mostly composed of C16 and C18 ω-hydroxyacids, polyhydroxyacids, epoxyacids, and α,ω-dicarboxylic acids. Suberin may be distinguished from cutin by higher contents of hydroxycinnamic acids, fatty alcohols, and saturated aliphatics with long chains (Beisson *et al.*, 2012).

Cutinases hydrolyze the plant leaf cuticle by cleaving the ester bounds of cutin (Dutta *et al.*, 2009). Therefore, cutinases belong to the esterase group, and more specifically to a class of serine esterases that contain the catalytic triad (serine, histidine and aspartate) with the active serine in the consensus sequence Gly-His/Tyr-Ser-X-Gly (Martinez *et al.*, 1994). Some fungal cutinases, such as the cutinase CcCUT1 of the fungus *Coprinopsis cinerea*, also exhibit the ability to degrade suberin (Kontkanen *et al.*, 2009). As lipolytic enzymes, cutinases have interesting properties for applications in various industrial processes (Carvalho *et al.*, 1999). For example, some cutinases exhibit the ability to degrade synthetic polyesters, such as polycaprolactone (Murphy *et al.*, 1996) and polyethylene terephthalate (PET) (Vertommen *et al.*, 2005; Eberl *et al.*, 2009; Ribitsch *et al.*, 2015).

Previous studies suggested that the bacterium *S. scabies* possesses the ability to degrade suberin. This pathogenic bacterium exhibits strong esterase activity in the presence of suberized tissues (Beauséjour *et al.*, 1999). Furthermore, a secretome analysis of *S. scabies* cultures grown in the presence of suberin revealed the presence of esterases, which are predicted to play a role in lipid metabolism (Komeil *et al.*, 2014; Beaulieu *et al.*, 2016). Among them, the Sub1 protein exhibits 33% identity with the cutinase CcCUT1 of the fungus *C. cinerea* (Komeil *et al.*, 2013). Interestingly, *sub1* gene expression was previously reported to be specifically induced in the presence of suberin (Komeil *et al.*, 2013). The cutinase from *Aspergillus oryzae* (PDB ID: 3GBS) is hallmarked by a central β-sheet of five parallel strands surrounded by ten α-helices (Liu *et al.*, 2009), as found for the predicted three-dimensional structure of the protein Sub1 (Supplemental Fig. S1). The model of the Sub1 protein also predicts the formation of two disulfide bonds (Cys31–Cys103; Cys178–Cys185) and a catalytic triad including residues Ser 114, Asp 182, and His 195 (Komeil *et al.*, 2013).

The main objectives of the present study were to produce the Sub1 protein, purify and characterize its enzymatic properties, and demonstrate that it functions as a polyesterase with the ability to degrade biopolymers, such as cutin and suberin, as well as the synthetic polyester PET.

## Materials and Methods

### Bacterial strains and culture conditions

An inoculum of *S. scabies* EF-35 (HER1481) was prepared in tryptic soy broth (10^8^ spores in 25 mL), as described previously (Komeil *et al.*, 2013). Cultures of *S. scabies* EF-35 were incubated with shaking (250 rpm) at 30°C. *Escherichia coli* strains DH5α (Invitrogen) and SHuffle T7 (New England Biolabs) were grown in LB medium supplemented where necessary with kanamycin (30‍ ‍μg‍ ‍mL‍^–1^) and were then incubated with shaking (250 rpm) at 37°C.

### Suberin and cutin preparation

A suberin-enriched potato periderm was obtained as previously described (Kolattukudy and Agrawal, 1974). The extracted material was dried under a hood, ground using a coffee mill, and stored at room temperature. To further remove residual polysaccharides in the potato periderm, this material was exposed to microbial degradation in the presence of *S. scabies* EF-35, as described by Beaulieu *et al.* (2016). The *S. scabies* inoculum (1 mL) was added to 50 mL of minimal medium consisting of a mineral solution (0.5‍ ‍g‍ ‍L‍^–1^ [NH_4_]_2_SO_4_, 0.5 g L^–1^ K_2_HPO_4_, 0.2 g L^–1^ MgSO_4_-7H_2_O, and 10 mg L^–1^ FeSO_4_-7H_2_O) and 1 g L^–1^ of the suberin-enriched potato periderm. After a 30-d incubation, 10 mL of fresh mineral solution and 200 μL of the *S. scabies* inoculum were both added to the culture and the incubation was extended for an additional 30 d. The culture was centrifuged at 3,450×*g* for 20‍ ‍min, and the pellet was resuspended in 100 mL of sterile water and then autoclaved for 15‍ ‍min. The suspension was washed with sterile water to remove bacterial cell debris. The resulting material (purified potato suberin) was dried at 50°C for 24 h. Cutin was isolated from apples following the protocol of Walton and Kolattukudy (1972).

### DNA extraction

Genomic DNA was isolated from 48-h bacterial cultures of *S. scabies* EF-35 using the GenElute Bacterial Genomic DNA Kit (Sigma-Aldrich) according to the manufacturer’s instructions. Plasmid DNA was isolated from 12-h *E. coli* cultures using the GenElute^TM^ Plasmid Miniprep Kit (Sigma-Aldrich) following the manufacturer’s instructions.

### Cloning of sub1 in *E. coli*

The *sub1* coding sequence, deprived of its signal peptide (GenBank accession number MK689853), was amplified by PCR from the genomic DNA of *S. scabies* EF-35 using the primers F-pET (5′-ATATCCATGGCCGCCTGCACGGACATCG-3′) and R-pET (5′-ATATCTCGAGTTAGATCTTGGTCGCGGCGAAGG-3′). The PCR mix contained 20 ng of DNA, 2.5 μL of *Taq* polymerase buffer, 0.5 μL of dNTPs (10‍ ‍mM), 0.5 μL (each) of forward and reverse primers (10 μM), and 0.125 μL of DNA *Taq* polymerase (New England Biolabs), in a total volume of 25 μL. PCR conditions consisted of 2‍ ‍min at 95°C followed by 30 cycles at 95°C for 30‍ ‍s, at 64°C for 1‍ ‍min, and at 68°C for 1‍ ‍min, with a final extension at 68°C for 5‍ ‍min. PCR was performed using the thermocycler T100 (Bio-Rad). Amplification products were migrated on a 1% agarose gel (Sambrook and Russell, 2001), purified from gels using the MinElute Gel Extraction Kit (Qiagen), and cloned into the pET-30a(+) vector (Novagen). The amplification product and cloning vector pET-30a(+) were both digested using the restriction enzymes *Nco*I and *Xho*I. Enzyme T4 DNA ligase (New England Biolabs) was used to ligate plasmid ends to amplicons following the manufacturer’s instructions. Ligation products were heat shock-transformed into competent cells of *E. coli* DH5α as per the manufacturer’s instructions (New England Biolabs). Bacteria were then incubated overnight on LB agar medium supplemented with kanamycin (30 μg mL^–1^). The plasmid insert was sequenced at a sequencing and genome genotyping platform (CHUL, University Laval, Quebec City, Canada). The plasmid pET-30a(+), with or without the *sub1* insert, was transformed into the expression host *E. coli* SHuffle T7, as previously described.

### Protein extraction

Cultures of *E. coli* SHuffle T7 carrying pET without or with the *sub1* gene insert (*E. coli* SHuffle T7-pET and *E. coli* SHuffle T7-pET-*sub1*) were incubated on LB agar medium supplemented with kanamycin. When OD_600_ reached 0.6–0.8, isopropyl β-D-1-thiogalactopyranoside (IPTG) was added to the culture (0 to 1.0‍ ‍mM, final concentration) and bacteria were incubated at 25°C for an additional 24 h. Cells were harvested by centrifugation (3,450×*g*) for 10‍ ‍min, pellets were washed twice with saline (NaCl 0.9%) and then resuspended in a buffer solution (50‍ ‍mM NaH_2_PO_4_ and 300‍ ‍mM NaCl, pH 8.0) supplemented with EDTA (2.5‍ ‍mM). The suspensions were sonicated on ice four times for 10‍ ‍s and centrifuged (3,450×*g*) at 4°C for 30‍ ‍min to remove cell debris. The supernatant was collected and successively passed through filters with pore sizes of 0.45 and 0.2 μm. The resulting protein solution was stored at 4°C.

### Purification of the protein Sub1

An affinity column Ni-NT cOmplete His-Tag purification column (Roche) was used to purify the protein Sub1 from the cytoplasmic fraction of *E. coli* SHuffle T7-pET-*sub1*, following the manufacturer’s instructions. Elution buffer A (50‍ ‍mM NaH_2_PO_4_, 300‍ ‍mM NaCl, pH 8.0), supplemented with different concentrations of imidazole (5 to 250‍ ‍mM), was used for column washing and His6-tagged protein elution. Protein fractions were subjected to sodium dodecyl sulfate-polyacrylamide gel electrophoresis (SDS-PAGE) migration along with the marker PageRuler™ Prestained Protein Ladder (Thermo Scientific), as described by Komeil *et al.* (2014). Proteins were stained with Coomassie brilliant blue R-250 (Bio-Rad; Lauzier *et al.*, 2008) and fractions containing purified Sub1 were pooled. This mixture was dialyzed in phosphate-buffered solution (PBS) to remove imidazole. Protein concentrations were measured according to Bradford (Bradford, 1976).

### Esterase activity of Sub1 on *p*-nitrophenyl esters

Esterase activity was assessed by spectrophotometrically measuring the absorbance of *p*-nitrophenol using the substrates *p*-nitrophenyl butyrate (C4), *p*-nitrophenyl octanoate (C8), *p*-nitrophenyl decanoate (C10), and *p*-nitrophenyl dodecanoate (C12) (Sigma-Aldrich). The molar extinction coefficient of *p*-nitrophenol in Tris-HCl (20‍ ‍mM, pH 7.5) at room temperature is 12,000‍ ‍M^–1^‍ ‍cm^–1^ at 420 nm. This enzymatic assay was performed as described previously (Komeil *et al.*, 2013) with slight modifications. In a 1.5-‍mL plastic cuvette, 20 μL of 100×-diluted Sub1 (60‍ ‍ng) was added to 970 μL of Tris-HCl (20‍ ‍mM, pH 7.5), with or without Triton X-100 (0.5%), and 10 μL of a 20‍ ‍mM *p*-nitrophenyl ester substrate (0.2‍ ‍mM final concentration). The absorbance at 420 nm of this reaction mix was measured at room temperature every 10‍ ‍s for 1‍ ‍min. The increase in absorbance of each sample was read against a blank without purified protein. One unit (U) was the amount of enzyme liberating 1 μmol of *p*-nitrophenol min^–1^ under the assay conditions. The *V*_max_ and *K*_m_ values of the enzyme Sub1 were assessed using the software GraphPad Prism7, according to the Michaelis-Menten equation, with different concentrations of the C4 substrate.

### Esterase activity of Sub1 on natural and synthetic polymers

Suberin and cutin were exposed to the enzyme Sub1 as follows. Suberin or cutin (10 mg) was added to 350 μL of Tris-HCl (20‍ ‍mM, pH 7.5) supplemented with 50 μL of the purified enzyme Sub1 (15 μg). Control assays (blanks) were made of suberin and cutin without the addition of the enzyme Sub1. The mixture was incubated at room temperature for 20 d. Colorimetric assays of free fatty acids released from the biopolymer were performed every 5‍ ‍d using a Free Fatty Acid Quantification Colorimetric/Fluorometric Kit (BioVision) according to the manufacturer’s instructions. A standard curve was prepared with palmitic acid to convert absorbance at 570 nm into fatty acid concentrations.

The hydrolyzing activity of Sub1 was also estimated on polyethylene terephthalate (PET) by measuring the amount of terephthalic acid (TA) released from PET according to Nimchua *et al.* (2008) with slight modifications. Assays were conducted in 2-mL tubes containing 10 mg of PET (ground granules, Sigma-Aldrich), 1 mL of Tris-HCl (20‍ ‍mM, pH 7.5), and 3 μg of the enzyme Sub1. In the first experiment, the effects of Triton on Sub1 performance were tested. Tubes, with or without Triton X-100 (0.5%), were incubated at 37°C and the concentrations of TA released in the incubation media were recorded after 10 and 15 d. Blank assays, in which the enzyme Sub1 was omitted, were used as controls. Tubes were then centrifuged (1‍ ‍min) and 50 μL of the collected supernatant was added to 350 μL of Tris-HCl (20‍ ‍mM, pH 7.5) into quartz cuvettes. Absorbance at 240 nm was measured to assess TA concentrations (using a standard curve). In another experiment, the stability of Sub1, using PET as a substrate, was assessed at 37 and 50°C over a 20-d period. The incubation medium contained Triton X-100 (0.5%) and the concentration of TA released in the reaction mix was measured every 5 d, as described above.

## Results

### Heterologous production of *Sub1*

**Fig. 1.**
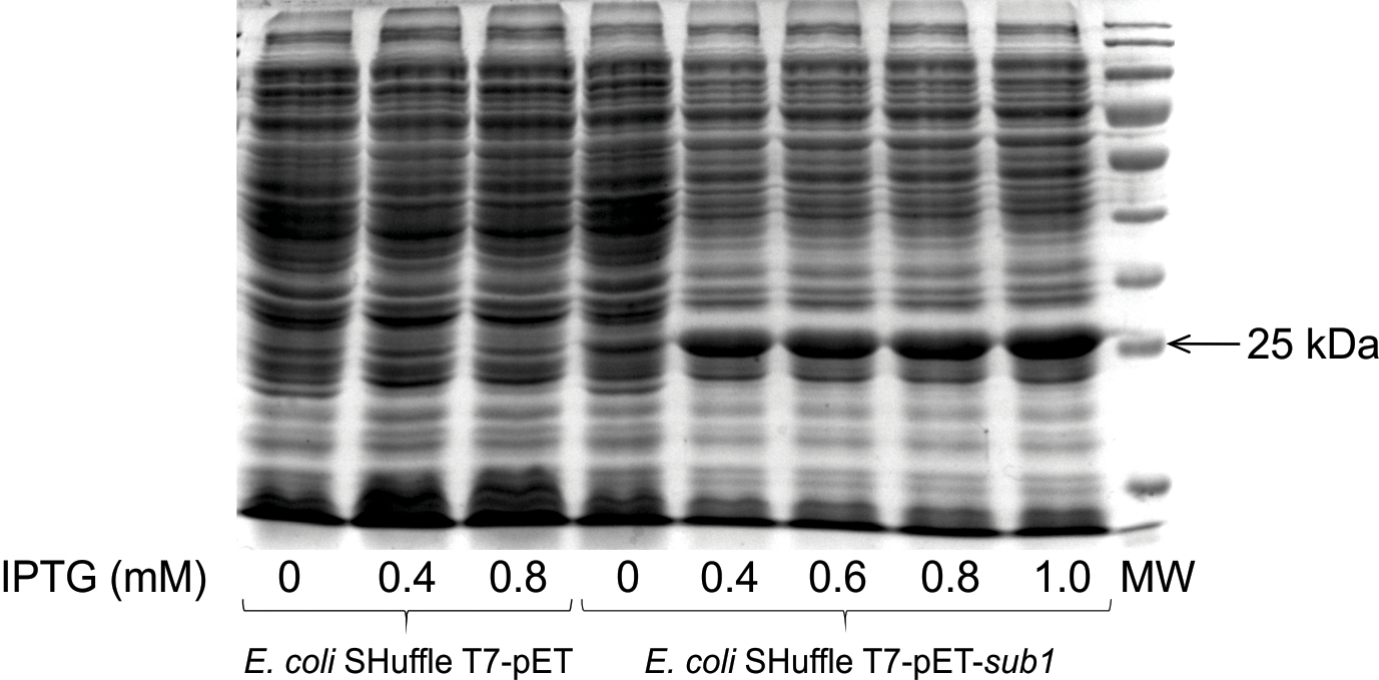
SDS-PAGE gel of the cytoplasmic extract obtained from pET-transformed *Escherichia coli* strain SHuffle T7, without (*E. coli* SHuffle T7-pET) or with (*E. coli* SHuffle T7-pET-*sub1*) the insert of the *sub1* gene, after induction with different concentrations of IPTG.

The *S. scabies sub1* gene was cloned into a pET expression vector (data not shown) and expressed in *E. coli* strain SHuffle T7. The cytoplasmic extract of *E. coli* SHuffle T7-pET-*sub1* was characterized by a thick and dense band of approximatively 25 kDa, which was absent from the protein profile of *E. coli* SHuffle T7-pET (Fig. 1). In *E. coli* SHuffle T7-pET-*sub1*, Sub1 was produced even in the absence of IPTG, while thicker protein bands were observed when *sub1* gene expression was induced by this compound (Fig. 1).

**Fig. 2.**
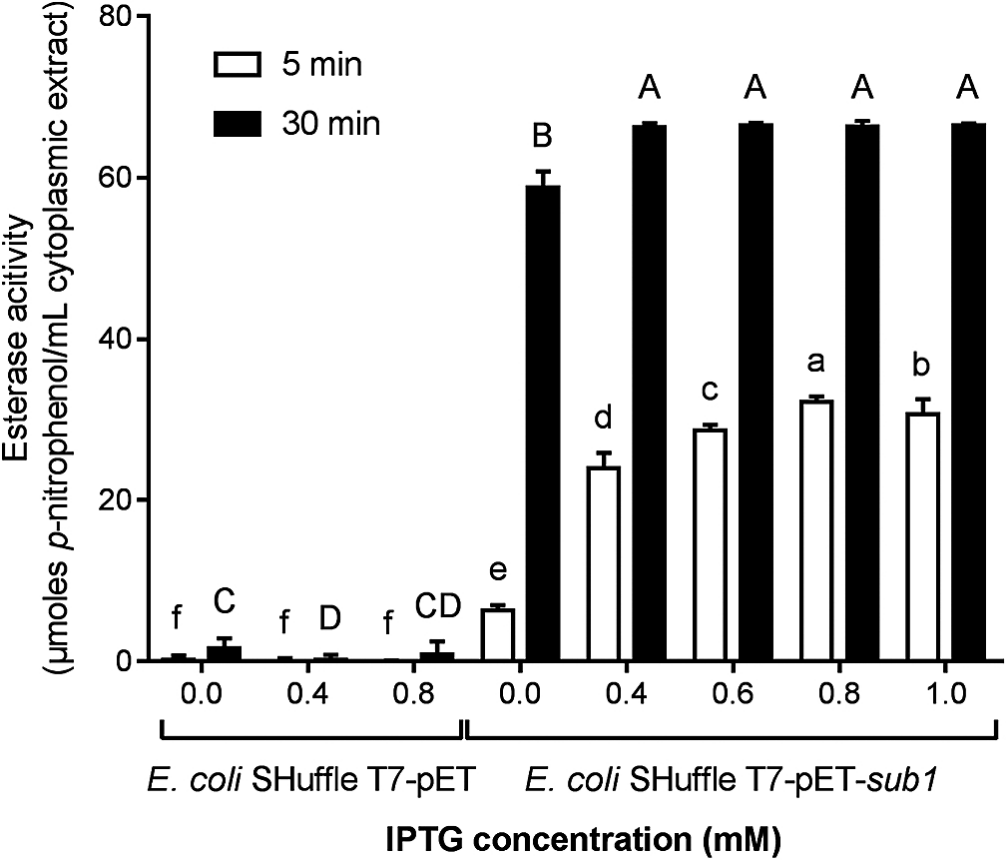
Esterase activity of cytoplasmic extracts from *Escherichia coli* SHuffle T7 transformed with plasmid pET without (*E. coli* SHuffle T7-pET) or with (*E. coli* SHuffle T7-pET-sub1) the *sub1* insert and exposed to various concentrations of IPTG. Activity is expressed as the concentration of *p*-nitrophenol released from *p*-nitrophenyl butyrate substrate in 5- and 30-min reactions. These results are the means of five replicates±SD. Bar values accompanied by the same lower case letter or upper case letter were not significantly different.

The cytoplasmic fraction from cultures of *E. coli* Shuffle T7-pET-*sub1* showed, in the absence of the induction with IPTG, that esterase activity on the C4 substrate (58.8 μmol mL^–1^) was significantly higher than that in control *E. coli* SHuffle T7-pET (1.6 μmol mL^–1^) after 30‍ ‍min of the incubation (Fig. 2). The esterase activity of *E. coli* SHuffle T7-pET-*sub1* was inducible with IPTG and was quickly observable in the reaction mix. After 5‍ ‍min of the incubation, the highest activity reached 32.2 μmol mL^–1^ when 0.8‍ ‍mM IPTG was added to the culture, while activity was 6.3 μmol mL^–1^ in the absence of IPTG (Fig. 2).

### Purification of the recombinant protein His-Sub1

**Fig. 3.**
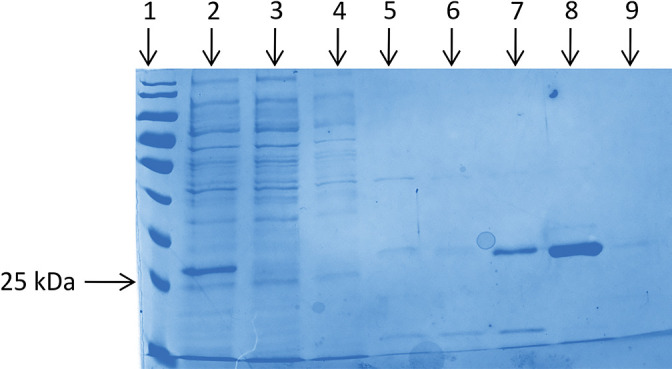
SDS–PAGE gel of cytoplasmic soluble proteins obtained from *Escherichia coli* transformed with SHuffle T7-pET-*sub1*, after fractionation on the affinity column (IMAC). Lane 1, molecular weight marker; lane 2, cytoplasmic extract; lane 3, flow-through; lane 4, proteins released after washing with buffer A; lanes 5 to 9, proteins released after washing with buffer A supplemented with 4, 5, 10, 50, or 200‍ ‍mM imidazole, respectively.

An affinity column Ni-NT (cOmplete His-Tag) was used to purify the recombinant protein Sub1 from the cytoplasmic fraction of *E. coli* SHuffle T7-pET-*sub1*. By comparing the migration profile of the cytoplasmic fraction and the flow-through (Fig. 3, lanes 2 and 3, respectively), the band corresponding to the recombinant protein His-Sub1 (25‍ ‍kDa) was present in the cytoplasmic fraction, while no band was present in the flow-through fraction, indicating that the Sub1 protein bound the column. The presence of imidazole in the elution buffer allows the elution of His-tag proteins from the column. At imidazole concentrations ranging between 4 and 10‍ ‍mM, the elution buffer released the majority of the contaminant proteins, while a low quantity of Sub1 was released with 10‍ ‍mM imidazole (lane 7, Fig. 3). A detectable quantity of Sub1 was eluted with no contaminant proteins when 50‍ ‍mM imidazole buffer was used (lane 8, Fig. 3). However, at 200‍ ‍mM imidazole, no recombinant protein was detected (lane 9, Fig. 3). A mass spectrometry analysis confirmed that the purified protein was Sub1 (data not shown). Purification efficacy was estimated by comparing the esterase activities (with 0.4‍ ‍mM *p*-nitrophenol butyrate as the substrate) of the cytoplasmic fraction and the purified protein. The esterase activity of Sub1 was 52-fold higher in the purified extract (23.0‍ ‍U‍ ‍mL^–1^) than in the cytoplasmic crude extract (0.4‍ ‍U‍ ‍mL^–1^).

### Esterase activity of the Sub1 protein on *p*-nitrophenyl esters

**Fig. 4.**
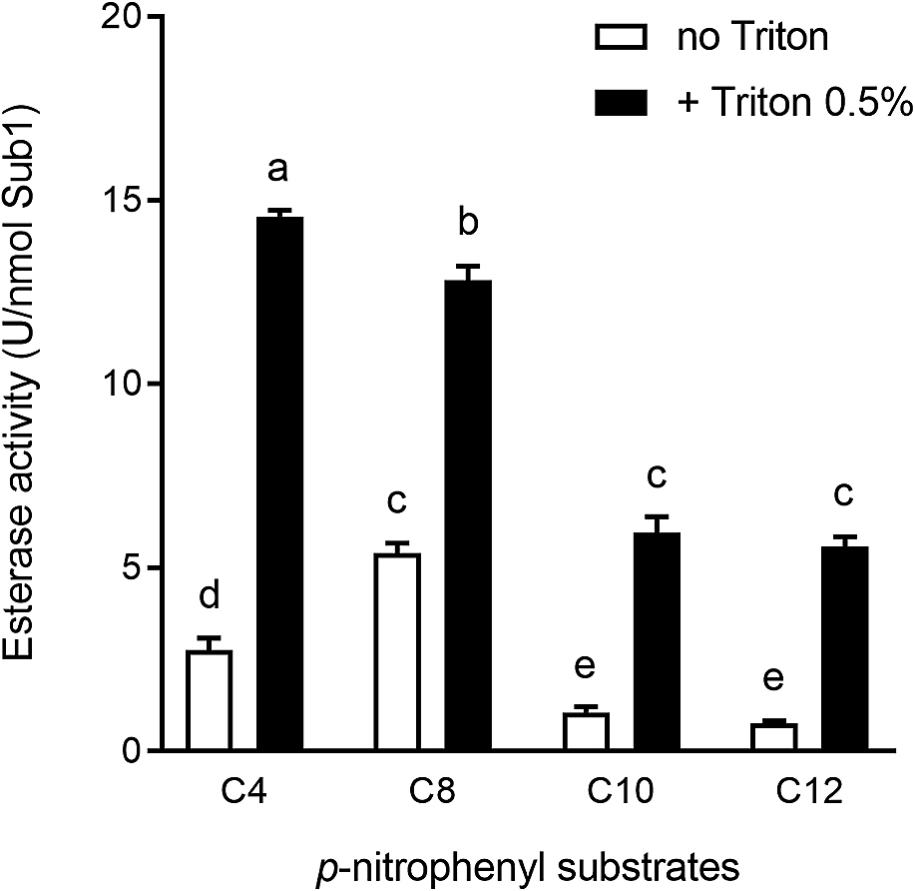
Esterase activity of the purified Sub1 enzyme using *p*-nitrophenyl substrates of different carbon chain sizes (C4, C8, C10, and C12) in the absence or presence of Triton X-100 (0.5%). Data shown are the mean±SD of three replicates. Bar values accompanied by the same letter are not significantly different.

**Fig. 5.**
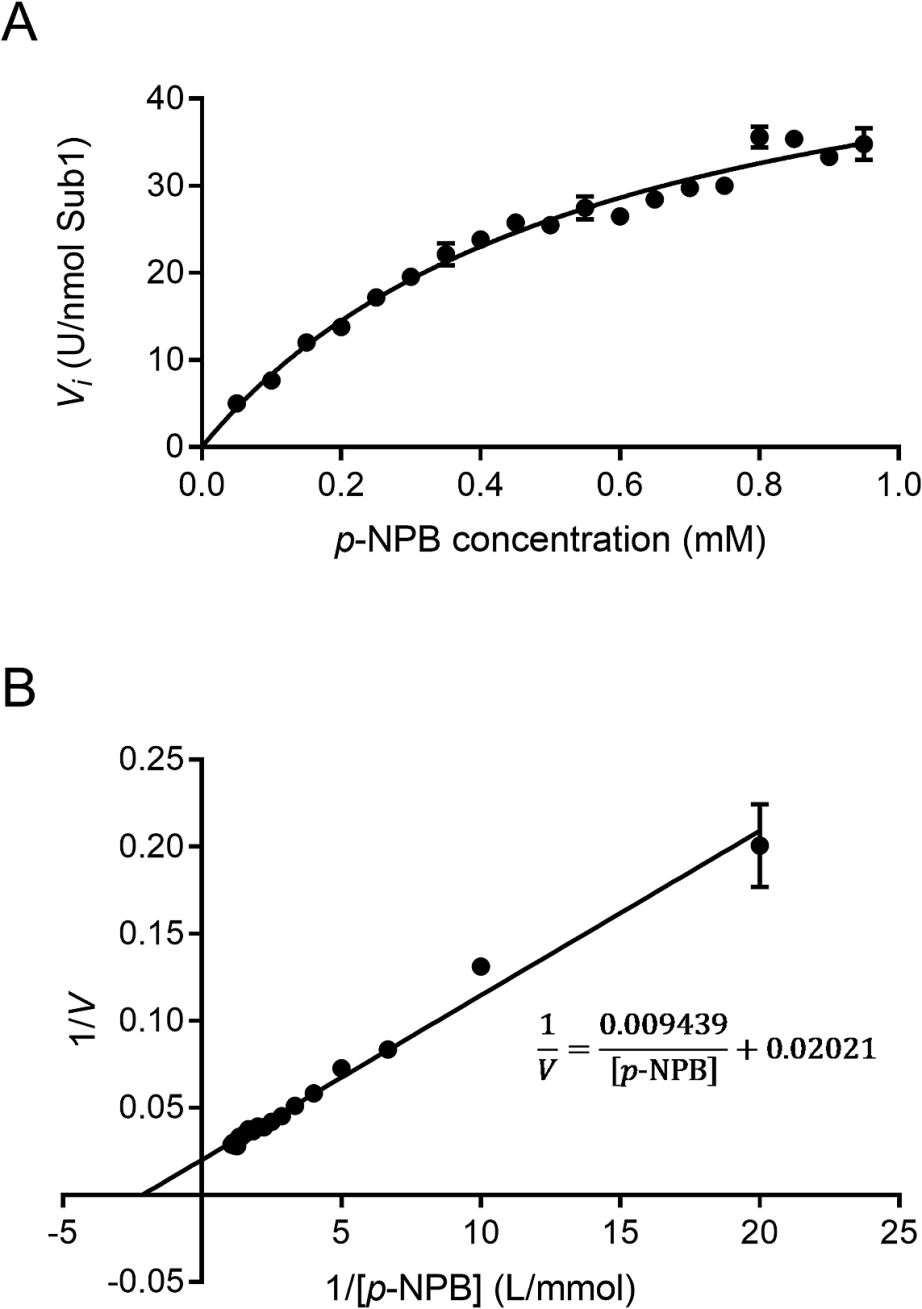
Effects of substrate (*p*-NPB) concentrations on the initial speed (*V*_0_) of the hydrolysis reaction of the esterase Sub1. (A) Michaelis-Menten kinetic and (B) Lineweaver-Burk plot. Data are the means±SD of three replicates.

The esterase activity of Sub1 on the *p*-nitrophenyl esters of varying carbon chain lengths was assessed in the presence and absence of Triton X-100. Independent of the presence of Triton X-100, Sub1 was more active on *p*-nitrophenyl butyrate (C4) and *p*-nitrophenyl octanoate (C8) than on *p*-nitrophenyl esters with longer carbon chains (C10 and C12, Fig. 4). The presence of Triton X-100 increased the esterase activity of the enzyme on all of these substrates (Fig. 4). The highest esterase activity (14.6 U nmol^–1^ or 616 U mg^–1^ Sub1) was obtained on the C4 substrate in the presence of Triton X-100. Thus, *V*_max_ and *K*_m_ values for esterase Sub1 were calculated using the latter substrate. As shown in Fig. 5, the initial velocity (*V*_0_) of the hydrolysis reaction increased when higher concentrations of the substrate *p*-nitrophenol butyrate (*p*-NPB) were used. According to the Michaelis-Menten equation, *V*_max_ was 55.8±2.0 U nmol^–1^ Sub1 (2,361±84.5 U mg^–1^ Sub1) and *K*_m_ was 0.57±0.04‍ ‍mM *p*-NPB.

### Effects of the Sub1 esterase on polymers

**Fig. 6.**
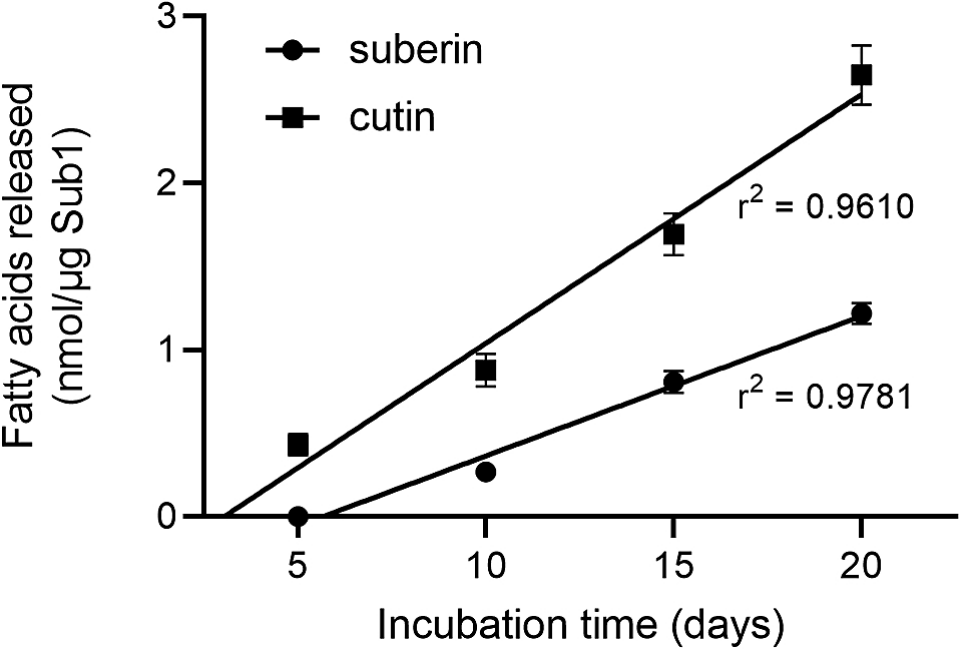
Degradation of cutin and suberin by enzyme Sub1 at room temperature over a 20-d period, as expressed by the release of fatty acids in the incubation medium. Data are the means±SD of four replicates.

Sub1 was shown to hydrolyze suberin and cutin, releasing 1.22±0.06 and 2.65±0.18 nmol of fatty acids (palmitic acid equivalent) μg^–1^ Sub1 from these polymers, respectively, after 20‍ ‍d of the incubation. The ability of Sub1 to degrade cutin and suberin appeared to be stable over the experimental time course at room temperature because the amounts of fatty acids released in the incubation medium correlated with time (*P*<0.0001; Fig. 6).

**Fig. 7.**
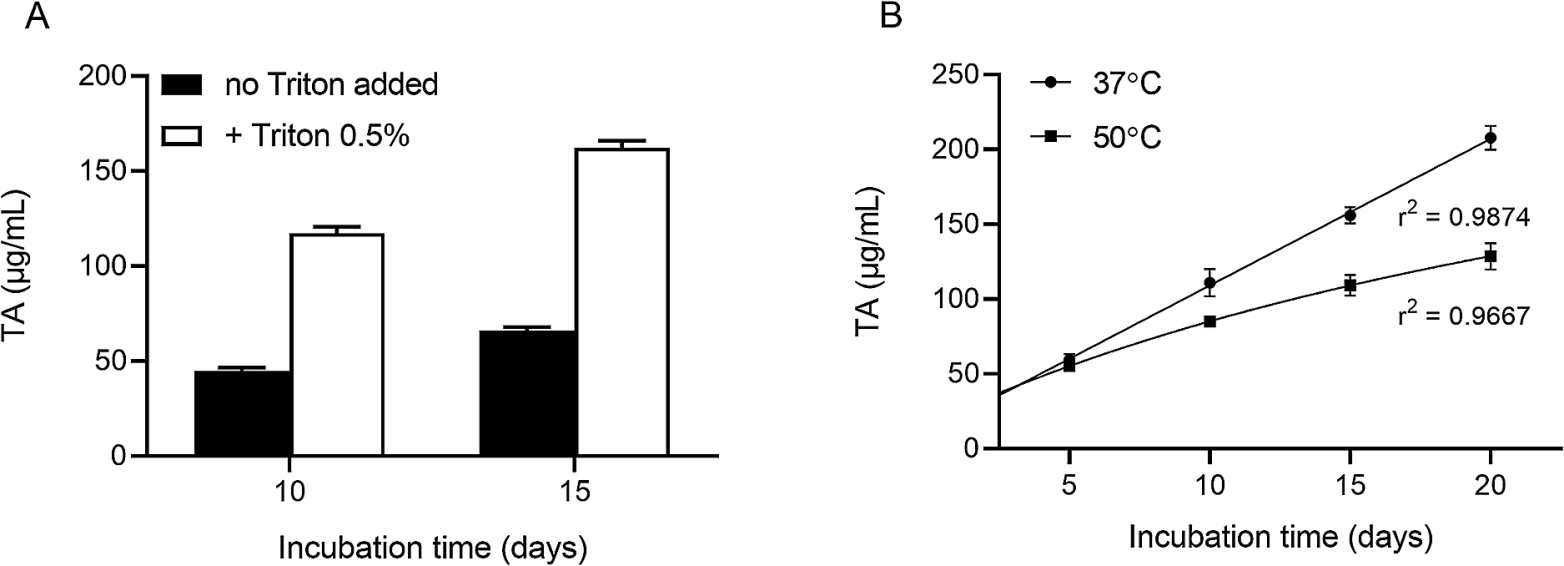
Concentrations of terephthalic acid (TA) released following the hydrolysis of ground particles of polyethylene terephthalate by 3 μg of the Sub1 enzyme. (A) Effects of the presence of Triton X-100 (0.5%) on Sub1 performance after 10 and 15‍ ‍d of incubation (at 37°C). (B) Sub1 enzymatic stability (in the presence of 0.5% Triton X-100) during 20‍ ‍d of incubation at 37 and 50°C. TA concentrations were measured every 5 d. Data are the means±SD of four replicates.

On the other hand, the release of TA showed that the Sub1 esterase also had the ability to hydrolyze the synthetic substrate PET. The addition of Triton X-100 to the reaction mix enhanced the hydrolysis of PET by *ca.* 2.6-fold (*P*<0.0001, *t*-test; Fig. 7A) after 10 and 15‍ ‍d of the incubation. The esterase activity of Sub1 on PET, in the presence of Triton X-100, increased over the incubation time because the amount of TA released during the degradation of PET correlated with the incubation time (*P*<0.0001; Fig. 7B). The enzyme Sub1 showed high stability at 37°C over the test period (20 d) because the concentration of TA released in the incubation medium linearly correlated with time (r^2^=0.9874). However, the stability of Sub1 at 50°C fit a non-linear curve (r^2^=0.9667).

## Discussion

*S. scabies* may colonize potato tuber surfaces and is able to directly penetrate potato cells (Loria *et al.*, 2003). Previous studies proposed that its entry into potato tuber tissues may be facilitated by the production of esterases that degrade suberin present in the potato periderm (McQueen and Schottel, 1987; Beauséjour *et al.*, 1999; Komeil *et al.*, 2013). Although suberin degradation has not yet been examined in detail, some fungal cutinases exhibit activity towards suberin (Kontkanen *et al.*, 2009). The protein Sub1 is part of the *S. scabies* secretome when this bacterium is grown in the presence of suberin (Beaulieu *et al.*, 2016) and the *sub1* gene is induced in the presence of suberin (Komeil *et al.*, 2013). This study predicted that Sub1 was a cutinase due to its high sequence homology with other cutinases of fungal origin. The present results confirm this prediction because Sub1 exhibited the ability to hydrolyze both cutin and suberin.

In the present study, the heterologous production of the *S. scabies* Sub1 protein was successfully achieved in *E. coli*. Other studies also reported the heterologous expression of bacterial esterases in *E. coli* (Chen *et al.*, 2008; Su *et al.*, 2013; Ribitsch *et al.*, 2015). The molecular weight of His-tagged Sub1 was estimated herein to be 25 kDa, which is consistent with the predicted molecular weight of mature Sub1 (18.7 kDa) plus the His-tag (4.9 kDa). Therefore, the molecular weight of Sub1 appears to be less than that of most bacterial cutinases, such as Tfu-0882 and Tfu-0883 from *Thermobifida fusca* (29 kDa; Chen *et al.*, 2008), but is closer to those of fungal plant pathogen cutinases, such as CcCUT1 from *C. cinerea* (18.8 kDa; Kontkanen *et al.*, 2009) and CutA from *Botrytis cinerea* (18 kDa; van der Vlugt-Bergmans *et al.*, 1997).

As reported with other cutinases, purified Sub1 also has the ability to hydrolyze *p*-nitrophenyl esters. Sub1 was more active on *p*-nitrophenyl esters with short carbon chains, *i.e.* *p*-nitrophenyl butyrate (*p*-NPB, C4) and *p*-nitrophenyl octanoate (C8), than on those with longer carbon chains (C10 and C12). Other microbial cutinases have also been reported to be more active on *p*-nitrophenyl esters harboring short fatty acid chains (Purdy and Kolattukudy, 1973; Kontkanen *et al.*, 2009). Using *p*-nitrophenyl butyrate as a substrate, the activity of Sub1 followed a typical Michaelis-Menten curve. The Sub1 enzyme showed affinity towards this substrate (*K*_m_=5.7 10^–4^ M), similar to two cutinases of *Fusarium solani* pv. *pisi* with *K*_m_ of 3.5 10^–4^ M and of 7.5 10^–4^ M, respectively (Kolattukudy *et al.*, 1981). Although streptomycetes such as *S. scabies* and filamentous fungi belong to different kingdoms, they exhibit similar lifestyles and often share the same ecological niches (Wösten and Willey, 2000). Their mycelia colonize various organic polymers and produce large amounts of extracellular enzymes to retrieve nutrients from these substrates. Therefore, similarities between Sub1 and fungal cutinases may reflect an adaptation to a similar lifestyle. Other *Streptomyces* extracellular enzymes, *e.g.* chitosanases from the GH75 family, are encoded by genes that are also mainly represented in fungal and actinobacterial genomes (Lacombe-Harvey *et al.*, 2018).

Sub1 has high similarity, at the amino acid level, to the cutinase CUT1 from *F. solani*, which is able to degrade suberin. Similar to CUT1, the present results indicated that Sub1 exhibits hydrolysis activity on suberin. However, Sub1 and CUT1 showed higher activity on cutin than on suberin, even though *S. scabies* and *F. solani* both infect and colonize potato tubers and are, thus, frequently in contact with potato suberin (Fiers *et al.*, 2012). This finding indicates that the presence of aromatic compounds in suberin also contributes to its recalcitrant nature (Faber, 1979). However, difficulties are associated with comparing the efficacy of Sub1 with fungal cutinases because the methods used to monitor cutinase activity differ between studies (van der Vlugt-Bergmans *et al.*, 1997; Kontkanen *et al.*, 2009; Chen *et al.*, 2013; the present results). To the best of our knowledge, Sub1 represents the first bacterial cutinase for which activity towards suberin has been demonstrated. Based on the ecological niche of this pathogen, in which potato tubers, but not the aerial part of the plant, are infected, suberin most likely represents an important substrate for Sub1 in the environment and, consequently, Sub1 may be designated as a suberinase. While the ability to degrade cutin has been shown to be important for the pathogenicity of various fungal plant pathogens (Feng *et al.*, 2011; Wang *et al.*, 2017), there is no evidence to show that the ability to degrade suberin represents an asset in the infection process of plant pathogens. The Sub1 protein does not appear to be an essential pathogenicity factor for *S. scabies* because the *sub1* gene has not been detected in other common scab-inducing *Streptomyces* species, such as *S. acidiscabies* (Komeil *et al.*, 2013), and its primary benefit may involve the degradation of refractory polymers. However, Sub1 may confer an advantage to *S. scabies* over other common scab-inducing species by facilitating direct penetration, tuber colonization, and persistence in potato tuber debris.

While Sub1 is conserved in only a few streptomycetes, it presents high homology not only with some fungal cutinases, but also with cutinase-like enzymes from animal pathogenic mycobacteria (Komeil *et al.*, 2013). These bacteria do not encounter cutin or suberin in their environment; however, cutinases were identified as multifunctional enzymes that act on phospholipids, polysorbates, triacylglycerols, and triolein (Schué *et al.*, 2010; Monu and Meena, 2016). As multifunctional enzymes, cutinases have a number of applications in industry (Carvalho *et al.*, 1998). In the present study, the effects of Sub1 on PET, a synthetic polyester that is widely used in the production of textiles, were tested. Sub1 was shown to degrade PET because the quantity of terephthalic acid released from the synthetic polymer depended on the enzyme concentration and increased over the incubation time. The enzyme Sub1 maintained its activity at 37°C for at least 20 d, showing that it is stable, as has been demonstrated for other cutinases (Dutta *et al.*, 2009). Due to their functional properties, cutinases are considered to be a link between esterases and lipases (Chahinian *et al.*, 2002). The addition of a non-ionic surfactant, such as Triton, into a reaction mix generally promotes the activity of lipases, but does not affect the activity of most cutinases. For example, the presence of Triton in the reaction mix increased the hydrolysis of polyester bis-(benzoyloxyethyl) terephthalate (3PET) by a lipase secreted by *Thermomyces lanuginosus*, whereas it did not exert any effect when the same substrate was exposed to cutinases secreted by *T. fusca* and *F. solani* (Eberl *et al.*, 2009). Triton increases the activity of lipases by promoting the opening of a peptide lid located over the active site of the enzyme; such a lid is not present in cutinases (Eberl *et al.*, 2009). As observed with most cutinases, the addition of Triton X-100 did not affect cutin hydrolysis by Sub1 (data not shown). Nevertheless, the hydrolysis of *p*-nitrophenyl esters and of PET by Sub1 was enhanced in the presence of Triton.

The present study established that the *sub1* gene of *S. scabies* encodes a protein acting as a suberinase. The versatility of Sub1 may also be considered for adoption in industrial applications. A cutinase-like enzyme has recently attracted global public attention. This enzyme, originally characterized in the bacterium *Ideonella sakaiensis* 201-F6, is able to degrade PET and has been reclassified as a PETase (Yoshida *et al.*, 2016). Thereafter, Austin *et al.* (2018) introduced modifications to the binding cleft of this enzyme and produced an engineered PETase with better plastic-degrading properties than the native enzyme. The current need for enzymes with the ability to degrade refractory polymers of anthropic origin makes further studies on the capabilities of Sub1 very relevant.

## Supplementary Material

Supplementary Material
