# Supplementary material for: Enzymatic Degradation of p-Nitrophenyl Esters, Polyethylene Terephthalate, Cutin, and Suberin by Sub1, a Suberinase Encoded by the Plant Pathogen Streptomyces scabies
Source: Microbes Environ. 2020 Feb 27;35(1):ME19086. doi: 10.1264/jsme2.ME19086 (PMC7104285; doi:10.1264/jsme2.ME19086)
Supplement: Supplementary file 1 — Supplementary Material [file 35_19086_s1.pdf]

Microbes Environ. Vol. 35, No. 1, 2020

Supplemental Material for

Enzymatic Degradation of *p*-Nitrophenyl Esters, Polyethylene Terephthalate, Cutin, and Suberin by Sub1, a Suberinase Encoded by the Plant Pathogen *Streptomyces scabies*

Raoudha Jabloune, Mario Khalil, Issam E. Ben Moussa, Anne-Marie Simao-Beaunoir, Sylvain Lerat, Ryszard Brzezinski and Carole Beaulieu

Département de biologie, Université de Sherbrooke, Sherbrooke (QC), J1K 2R1, Canada

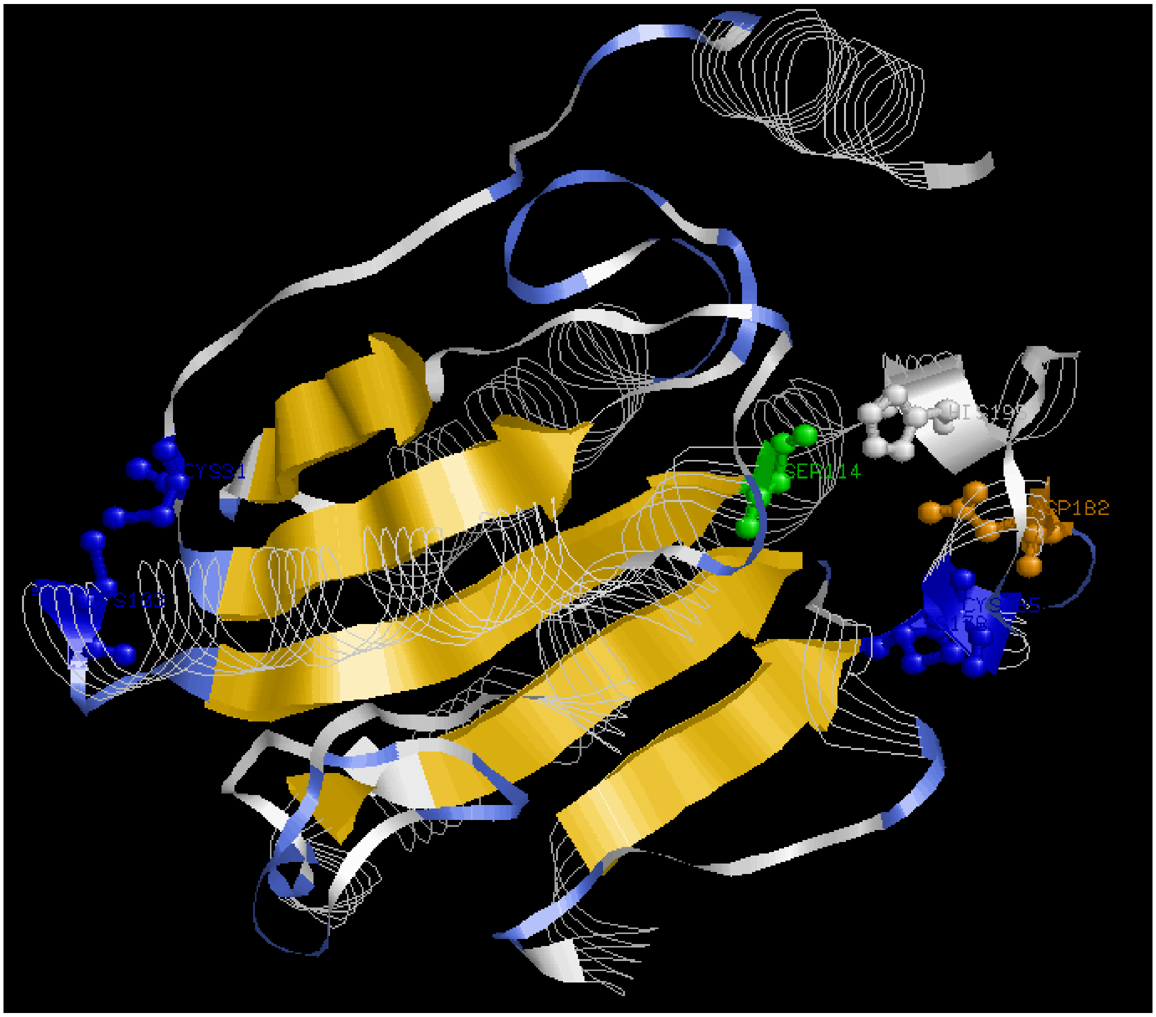

Supplemental Figure S1. Three-dimensional structural model of the Sub1 protein. The structural model was obtained submitting the deduced amino acid sequence of Sub1 (devoided of the signal peptide segment) to the ESyPred3D server (Lambert *et al*., 2002). The graphic was executed with the RasMol program (Sayle and Milner-White, 1995). Color legend: yellow, β-sheets; white, α-helices; blue, disulfide bonds; and green-brown-white, catalytic triad.

References

Lambert, C., Leonard, N., De Bolle, X., and Depiereux, E. (2002) ESyPred3D: prediction of proteins 3D structures. *Bioinformatics* **18**: 1250–1256.

Sayle, R., and Milner-White, J. (1995) RasMol: Biomolecular graphics for all. *Trends Biochem Sci* **20**: 374–376.
